# Research Progress in Plant Beneficial Fungi-Mediated Alleviation of Drought Stress in Crops

**DOI:** 10.3390/jof12030188

**Published:** 2026-03-05

**Authors:** Xiao-Han Wu, Qing-Yun Gu, Chen-Yu Ma, Wei Zhang, Chuan-Chao Dai

**Affiliations:** Jiangsu Key Laboratory for Pathogens and Ecosystems, Jiangsu Engineering and Technology Research Center for Industrialization of Microbial Resources, College of Life Sciences, Nanjing Normal University, Nanjing 210023, China; 18651676603@163.com (X.-H.W.); guqy0214@163.com (Q.-Y.G.); macy0422@126.com (C.-Y.M.)

**Keywords:** beneficial fungi, crop, drought stress, rhizosphere microbiome, assembly mechanisms

## Abstract

Climate change has emerged as a major global concern and has substantially intensified the occurrence of abiotic stresses in plants. Among the abiotic constraints limiting crop production, drought stress is regarded as one of the most severe and pervasive challenges. To this end, developing efficient and sustainable strategies to mitigate drought has become an urgent priority in agricultural research. Current approaches to improving drought tolerance mainly include optimizing irrigation management, applying chemical regulators, and breeding drought-resistant cultivars. However, these strategies often suffer from high input costs, limited durability of effects, potential environmental risks, or restricted regional applicability, making it difficult to achieve long-term and stable drought mitigation. In recent years, a growing body of evidence has indicated that rhizosphere microorganisms play pivotal regulatory roles in plant drought adaptation, with beneficial fungi being particularly important. Nonetheless, the key processes and mechanisms by which microbiomes mediate crop adaptation to drought need to be elucidated systematically. In this review, we synthesize recent advances in the field and, against the backdrop of increasingly severe global drought, summarize the major impacts of drought stress on crop growth and physiological processes. We further systematically synthesize the key mechanisms by which beneficial fungi alleviate drought stress in crops. Finally, we outline future research directions to deepen our understanding of rhizosphere–crop–microbe interaction networks and to provide a theoretical basis for developing beneficial fungus-centered microbial biofertilizers and microbiome-mediated strategies to enhance crop drought resilience.

## 1. Introduction

Against the backdrop of global climate change, the frequency of agrometeorological disasters, including drought, has continued to rise [1,2]. In recent years, drought has markedly constrained plant growth and yield, making drought tolerance a key determinant of crop productivity and persistence [3,4]. As one of the most destructive environmental stresses, drought poses a severe threat to agricultural production as well as to human and livestock livelihood security [5,6]. Drylands, comprising semi-arid, arid, and hyper-arid regions, cover approximately 41% of the Earth’s land surface and have expanded considerably in recent decades [7]. In this context, water deficits not only markedly reduce crop yield and quality but also exert profound and often difficult-to-reverse negative impacts on agroecosystems [8,9]. Globally, major crops such as wheat, maize, rice, and cotton have long been exposed to recurrent drought stress, commonly exhibiting impaired growth and development, increased disease incidence, and substantial yield losses, thereby imposing serious pressure on agricultural production systems [10,11,12,13,14]. One study reported that drought is projected to cause the most severe production losses in soybean, with yield reductions reaching 3.6%, whereas rice is projected to be the least affected among the crops assessed [4]. Approximately 55 million people worldwide are currently affected by drought, and by 2030, the number of people at risk of displacement due to drought may increase to around 700 million, leading to enormous economic losses [15,16]. Therefore, developing scientifically sound and effective drought-mitigation strategies has become an urgent priority for advancing green agriculture and achieving sustainable agricultural development.

Drought stress first manifests in crops as inhibited morphological development and pronounced yield loss [15,17,18]. In aboveground tissues, drought commonly causes leaf wilting and drooping, accompanied by reduced leaf size, increased leaf thickness, and a shift in leaf color from deep green to gray-green or yellow [19,20]. As stress intensifies, marginal scorching and leaf rolling become more severe [21,22]. Belowground, severe drought markedly suppresses normal root development, leading to substantial reductions in total root length and total root surface area; under severe conditions, it may even induce necrosis of root tip cells [23]. These changes weaken the ability of crops to efficiently acquire water and nutrients, particularly as soils progressively harden, ultimately resulting in yield reduction. For example, across crops such as rice, peanut, wheat, cotton, potato, maize, and sugar beet, water deficit generally decreases yield in these crops [24,25,26,27,28,29,30]. Under drought conditions, wheat and rice yields decline by 27.5% and 25.4%, respectively, while peanut pod yield losses can be as high as 85% [25,31]. In addition to morphological alterations, drought can also induce a suite of physiological adaptive responses in crops; however, when these regulatory mechanisms are insufficient to counteract stress, metabolic perturbations and cellular damage ensue [32]. As the fundamental process underpinning carbon fixation and normal plant growth and development, photosynthesis is often markedly inhibited [33]. At the early stage of drought, plants typically reduce transpiration by closing stomata, thereby limiting water loss and improving water-use efficiency [34]. Under prolonged or severe drought, however, intercellular CO_2_ concentration in leaves remains stable or even increases, indicating that the decline in photosynthesis is no longer primarily stomatal-limited. At this stage, the photosystem II (PSII) reaction centers are damaged and the electron transport chain is disrupted, leading to suppressed photosynthetic electron transport [15,35]. In this process, excess excitation energy, decreased chlorophyll content, structural impairment of the photosynthetic apparatus, and excessive accumulation of reactive oxygen species (ROS) may occur, ultimately causing irreversible injury and even plant death [36]. For instance, severe drought markedly reduces stomatal conductance and net photosynthetic rate in peanut and induces elevated ROS levels in roots, thereby inhibiting plant growth. Sustained ROS accumulation further accelerates membrane lipid peroxidation, decreases antioxidant enzyme activities, and compromises membrane integrity [37,38]. Malondialdehyde (MDA), a terminal product of lipid peroxidation, is therefore commonly used as an important indicator of stress severity [39]. In rice, drought-induced reductions in chlorophyll content and CO_2_ assimilation capacity are accompanied by increased MDA accumulation, resulting in irreversible yield penalties [40].

Conventional drought-mitigation approaches, such as breeding drought-tolerant cultivars, applying chemical regulators, and optimizing irrigation management, can alleviate drought stress to some extent, but they are often constrained by long breeding cycles, potential environmental risks, or high implementation costs [41,42]. In recent years, beneficial microbes have been increasingly explored as environmentally friendly, cost-effective, and potentially sustainable tools to promote crop growth under drought conditions (e.g., in peanut, wheat, maize, and sorghum). This approach has rapidly become a research hotspot in agricultural drought-tolerant research [16,43,44,45]. The rhizosphere microbiome refers to the entire assemblage of microorganisms residing in the plant rhizosphere and interacting closely with the host, encompassing bacteria, fungi, protists, and viruses [46,47]. Among these, the distinctive growth habits and metabolic traits of beneficial fungi enable them to establish stable mutualistic associations with plants and play pivotal roles in promoting plant growth and enhancing drought tolerance [48,49,50]. Beneficial fungi mainly include endophytic fungi and mycorrhizal fungi, which can rebalance hormone homeostasis and enhance water uptake through extensive hyphal networks, thereby increasing root hydraulic conductivity, improving plant water status, and reducing the adverse impacts of drought [51,52,53]. In addition, beneficial fungi can strengthen the plant antioxidant system, promote osmotic adjustment, and improve nutrient acquisition efficiency under drought conditions [54,55]. Collectively, these coordinated responses help plants maintain growth and stress tolerance under water-limited environments. Nevertheless, given the high complexity of the rhizosphere–plant–microbe system, a key unresolved challenge is how to accurately identify and select beneficial fungal taxa that confer drought tolerance while being able to colonize field environments stably and persistently over time.

Existing studies indicate that, under drought conditions, the application of a single fungal strain often suffers from inconsistent performance and/or limited persistence [56]. In contrast, beneficial microbial communities formed by rationally combining fungi with functionally distinct microorganisms can act synergistically at the community level and provide plants with more comprehensive ecological services [57,58,59]. Beneficial microbial communities have been widely applied across multiple research fields, which has facilitated community-level dissection of microbiome assembly processes and plant–microbe interactions [60,61,62]. Meanwhile, this approach has also demonstrated clear advantages in improving nutrient acquisition efficiency, productivity, and drought tolerance [16,63,64]. Accordingly, microbial communities are widely regarded as a promising technological avenue for advancing sustainable agriculture [65,66]. Nevertheless, their intrinsic structural complexity means that the design of microbial communities has often not fully accounted for the potential interactions among member strains. To this end, this review is set against the backdrop of current drought conditions and systematically elucidates how drought stress affects crop growth and physiological processes. With beneficial fungi as the focal point, we highlight the specific mechanisms by which beneficial microbes alleviate drought stress in crops. Beneficial fungi can enhance crop drought tolerance through two pathways: direct mechanisms (modulating physiological and molecular responses) and indirect mechanisms (reshaping the rhizosphere microbial community). Meanwhile, we emphasize the key roles of rhizodeposits, plant immune regulation, and microbe–microbe interactions in the beneficial fungus-mediated restructuring of the rhizosphere microbiome. Finally, by linking these mechanisms to enhanced crop drought performance, we discuss the translational potential of fungi in agricultural management, providing a theoretical basis for leveraging beneficial microbes to sustain crop yield under drought and to promote long-term agricultural sustainability.

## 2. Mechanisms by Which Plant Beneficial Fungi Alleviate Drought Stress in Crops

### 2.1. Direct Mechanisms: Modulation of Physiological and Molecular Responses

Beneficial fungi can enhance crop drought tolerance through multiple direct mechanisms, including improving root water uptake and soil water retention, maintaining plant hormone homeostasis, increasing antioxidant enzyme activities, strengthening osmotic adjustment, and regulating drought-responsive molecular regulatory networks (Table 1).

#### 2.1.1. Root Water Uptake Enhancement and Soil Water Retention

Under drought stress, one of the most direct contributions of beneficial fungi is to help roots absorb more water by modulating root hydraulic conductivity and improving soil water retention properties [93]. In particular, plants colonized by arbuscular mycorrhizal fungi (AMF) often exhibit higher root hydraulic conductivity and symplastic flow, possibly due to the upregulated expression of root aquaporins, thereby enabling plants to take up more water [77,94]. Consistently, under water deficit, non-mycorrhizal roots show reduced water permeability and cellular hydraulic conductivity, whereas mycorrhizal roots tend to maintain levels comparable to those under non-water-stressed conditions [95]. Beyond root-level regulation, AMF develop extensive extraradical hyphal networks that penetrate fine soil pores, absorb water, and deliver it to root tissues through continuous hyphal conduits, thereby alleviating plant water deficits under drought [96,97]. Owing to the partial hydrophobicity of hyphal cell walls, water is less prone to loss during translocation, which helps maintain relatively stable water-conducting routes in dry soils and enables plants to exploit water resources that are otherwise difficult to access [98]. Beyond improving water acquisition, beneficial fungi can enhance soil water retention by reshaping soil structure. Interactions between fungal hyphae and soil particles promote the formation of water-stable aggregates, thereby increasing soil water-holding capacity and reducing erosion [50]. As a dominant drought-adaptive fungal group, AMF further contribute to aggregate stabilization by releasing hydrophobic organic substances into the soil matrix, such as polysaccharides, glomalin-related soil protein (GRSP), and mucilaginous materials, thereby promoting carbon sequestration and enhancing aggregate persistence [99,100]. For example, potassium-sequestering glomalin produced by *Gigaspora margarita* can enhance peanut drought tolerance and pod yield [67]. Moreover, beneficial microbial communities can further improve crop water status through complementary functions. AMF strengthen rhizosphere structure and promote aggregate formation via extraradical hyphae and glomalin, thereby increasing soil water-holding capacity and providing a more stable water supply to the host during drought. Meanwhile, bacteria can synthesize drought-associated compounds such as exopolysaccharides; when combined with AMF, these traits confer superior soil water retention and markedly enhance host drought resilience, as demonstrated in multiple systems [82,101,102]. Collectively, plant–microbe interactions play central roles in improving root water uptake and soil water retention, which together contribute to enhanced plant drought tolerance.

#### 2.1.2. Plant Hormone Homeostasis Regulation

Plant hormones (e.g., indole-3-acetic acid, IAA; abscisic acid, ABA; brassinosteroids, BRs; cytokinins, CKs; ethylene, ET; gibberellins, GAs; jasmonic acid, JA; and strigolactones, SLs) are key regulators of plant growth and development [103]. Plant hormones reprogram drought-responsive gene expression and trigger a cascade of physiological adjustments, ultimately enabling plants to cope with water deficit through drought escape, avoidance, and tolerance strategies [80,104]. Accumulating evidence indicates that under drought stress, colonization by beneficial fungi can markedly influence hormone biosynthesis and homeostasis [78,90,105,106,107]. For example, Ruiz et al. reported that, under drought conditions, AMF induced strigolactone biosynthesis in the host and increased growth and photochemical performance in tomato, elevated ABA levels, and upregulated ABA-related marker genes, thereby enhancing drought tolerance [69]. In addition, inoculation with *Phomopsis liquidambaris* B3 was shown to promote IAA biosynthesis in rice and consequently affect plant growth [108]. At the molecular level, colonization by certain beneficial fungi can upregulate *LOXD*, thereby promoting JA biosynthesis, while enhancing ABA production via the induction of *NCED3* [109,110,111]. Fungal-plant symbioses may also systemically rebalance hormonal networks, coordinating the levels of IAA, ABA, and CKs, among others. For instance, inoculation of maize with AMF alleviated drought stress by increasing IAA and ACC contents and elevating the IAA/ABA, IAA/ZT and IAA/ACC ratios [81]. Moreover, beneficial fungi can also interact with other rhizosphere microorganisms to form microbial communities, which often confer stronger drought-mitigation effects than single fungal strains [64,88]. Within such microbial communities, beneficial fungi can modulate hormone biosynthesis, transport, metabolism, and function in the host and in partner microbes, thereby shaping host growth and drought tolerance [79]. For example, Wu et al. found that, under drought, the endophyte *Ph. liquidambaris* B3 influenced salicylic acid (SA) biosynthesis in peanut roots; SA subsequently promoted the recruitment and enrichment of the drought-adaptive AMF *Claroideoglomus etunicatum* in the peanut rhizosphere, thereby improving drought tolerance [38]. In another case, colonization by *Funneliformis mosseae* stimulated L-tryptophan production in soybean root exudates. Acting as a signaling molecule, L-tryptophan activated a cross-kingdom signaling cascade that upregulated genes encoding IAA methyltransferase, thereby inducing IAA biosynthesis in the exudates of *Pseudomonas putida* KT2440 [76]. Collectively, plant–microbe interactions and the elucidation of hormone-associated signaling links among microbial partners represent important directions for enhancing host drought tolerance.

#### 2.1.3. Antioxidant Enzyme Activity Elevation

Plants possess an antioxidant network composed of enzymatic and non-enzymatic defense systems [112,113]. The non-enzymatic component mainly relies on glutathione (GSH) and the ascorbate–glutathione (AsA-GSH) cycle to maintain cellular redox homeostasis [114]. Within the enzymatic system, superoxide dismutase (SOD) represents the first line of defense against superoxide anion stress. In general, Mn/Fe-SODs are predominantly localized in mitochondria, whereas Cu/Zn-SODs mainly occur in chloroplasts and the cytosol. Catalase (CAT), together with ascorbate peroxidase (APX) and glutathione reductase (GR), acts coordinately to remove intracellular hydrogen peroxide (H_2_O_2_) via the AsA–GSH cycle; peroxidase (POD) can additionally oxidize phenolic compounds [115,116]. Drought stress promotes excessive production of reactive oxygen species (ROS) and causes oxidative damage, and enhanced antioxidant activity is closely associated with improved drought tolerance [117]. Within this oxidative-stress framework, interactions between beneficial fungi and their host plants have emerged as an important determinant of plant antioxidant capacity, particularly under drought conditions [38,68,79]. A substantial body of evidence indicates that, relative to non-inoculated controls, plants colonized by beneficial fungi typically exhibit higher antioxidant enzyme activities and lower accumulation of oxidative-damage markers [80,85,87]. For instance, in maize, AMF symbiosis can mitigate drought-induced non-systemic oxidative injury by reducing ROS levels and strengthening antioxidant defenses [80]. These physiological and biochemical shifts are further supported by transcriptional regulation and metabolic reprogramming. Studies have shown that, under drought, inoculation with beneficial fungi can also upregulate the expression of antioxidant enzyme-encoding genes in host plants. For example, under drought stress, the expression levels of *PtMnSOD*, *PtCAT1*, and *PtPOD* in orange leaves are significantly increased, thereby enhancing antioxidant enzyme activities and improving ROS scavenging capacity [72]. This suggests that fungal regulation of plant antioxidant capacity not only involves post-translational activation of existing enzymes but also extends to upstream control of gene expression. In addition, AMF can coordinately regulate antioxidant defenses and other stress-resistance responses by modulating the MAPK signaling pathway and hormone-mediated signaling [102]. Under drought conditions, microbial communities formed by beneficial fungi in association with other rhizosphere microorganisms often confer stronger antioxidant protection than single-strain inoculation. For example, in *Myrtus communis* L., co-inoculation with *Funneliformis mosseae*, *Rhizophagus irregularis* and *Pseudomonas fluorescens* increases both enzymatic and non-enzymatic antioxidant pools, thereby substantially enhancing drought tolerance [75]. RNA-sequencing analyses further indicate that such microbial communities activate multiple metabolic pathways associated with redox homeostasis (e.g., the pentose phosphate pathway that supplies NADPH for antioxidant reactions, as well as pathways related to methionine and sulfur metabolism) and upregulate a broad set of antioxidant enzyme-encoding genes [118]. Collectively, drought-protective microorganisms can coordinately modulate enzymatic and non-enzymatic antioxidant systems, thereby strengthening the redox buffering capacity of plants under water-limited conditions.

#### 2.1.4. Osmotic Adjustment Enhancement

Osmotic adjustment is a key strategy to improve plant drought tolerance [119,120]. Osmoregulatory substances mainly fall into two categories: inorganic electrolytes (e.g., K^+^) and organic osmolytes (e.g., soluble carbohydrates, polyols, nitrogen-containing compounds, and other compatible solutes) [121]. Under drought stress, beneficial fungi can markedly increase the levels of these osmoregulatory compounds in their hosts, thereby lowering cellular osmotic potential, maintaining turgor pressure and water status, and ultimately alleviating drought-induced injury [68,80,122,123]. For example, in peanut, inoculation with the *Ph. liquidambaris* B3 significantly increased K^+^, soluble sugar, and soluble protein contents in both leaves and roots [38]. Regarding soluble sugar composition, *Funneliformis mosseae* significantly upregulated the expression of *PtAI*, *PtNI*, and *PtSPS* in drought-stressed orange roots, promoting sucrose cleavage and consequently increasing the contents of glucose and fructose [73]. Meanwhile, AMF increased trehalose levels in orange roots only under drought conditions, suggesting a stress-dependent (“on-demand”) regulatory pattern. This shift was associated with AMF-induced upregulation of *PtTPS1* and repression of *PtTRE1–5* [74]. Moreover, under drought conditions, microbial communities often exert stronger osmotic-adjustment benefits than any single inoculant [63,79,82]. For instance, Compared with inoculation with either microbial agent alone, co-inoculation with *Glomus intraradices* and *Azospirillum brasilense* resulted in the highest proline content in rice, thereby conferring greater drought tolerance [87]. Co-inoculation of *Aspergillus violaceofuscus* and *Bacillus licheniformis* also increased host K^+^ contents, thereby enhancing drought tolerance in tomato [71]. Collectively, beneficial microorganisms can facilitate osmotic adjustment and water retention by elevating osmoregulatory compounds, helping crops maintain cellular function and growth under drought stress.

#### 2.1.5. Molecular Regulatory Mechanism Modulation

Drought-induced plant responsive factors can be broadly classified at the gene level into two categories: (1) function-related genes, which directly participate in stress adaptation processes (e.g., aquaporins, AQP) [80,84], and (2) regulatory genes, which primarily mediate signal transduction and transcriptional regulation (e.g., stress-related transcription factors and components of Ca^2+^ signaling pathways) [102,124,125]. Under drought conditions, inoculation with beneficial fungi often induces the upregulation of multiple drought-responsive genes in host plants, with one common outcome being enhanced water uptake and transport capacity [70,78,79,90,91,126,127]. AMF can upregulate the expression of specific root AQP genes, thereby improving water transport efficiency, which may represent one mechanism by which AMF enhance plant drought tolerance [128]. For example, in *Robinia pseudoacacia*, inoculation with *Rhizophagus irregularis* significantly increased the expression of eight plasma membrane intrinsic proteins (PIPs) genes in roots and enhanced the expression of the AMF hyphal aquaporin gene *GintAQP1*, which in turn increased biomass and improved tissue water status and photosynthesis, suggesting that AQP-related regulation may contribute to improved drought tolerance [83]. In addition to AMF, other beneficial endophytic fungi can also reshape drought-induced gene expression patterns in crops. In rice, colonization by *Trichoderma harzianum* can alter the drought-induced expression patterns of genes related to *AQU* and *DHN*, thereby helping crops maintain productivity under water deficit [86]. In wheat, Yue et al. reported that co-inoculation with *Mortierella alpina* and *Epicoccum nigrum* led to the upregulation, to varying extents, of multiple stress-related genes, including *CIPK9* and *PP2C30* [89]. *Rhizophagus irregularis* inoculation upregulated the expression of *RiCPSI* and *RiCARI* in *Medicago sativa*; these molecular adjustments collectively contribute to enhanced drought tolerance in the AMF–plant symbiosis [92]. Furthermore, co-inoculation of plant beneficial fungi with other soil microbes can enhance host drought resilience through additive molecular responses. For instance, in maize, co-inoculation of *R. irregularis* with the phosphate-solubilizing bacterium *Bacillus megaterium* upregulated aquaporin-related genes, including *ZmPIP1;3*, *ZmTIP1;1*, and *GintAQPF1*, thereby increasing root hydraulic conductivity and improving drought tolerance [82]. Collectively, these studies indicate that beneficial microbiomes can promote the maintenance of plant water status and physiological functions under drought by modulating the expression of both function-related and regulatory genes.

### 2.2. Indirect Mechanisms: Reshaping the Rhizosphere Microbial Community

In addition to the direct drought-alleviating effects described above, beneficial fungi can also indirectly enhance crop drought tolerance by reshaping the rhizosphere microbiome. For example, following AMF colonization, improved drought tolerance in maize is closely associated with shifts in the structure of rhizosphere bacterial communities [129]; under drought conditions, inoculation with the *Ph. liquidambaris* B3 can enrich drought-functional AMF *C. etunicatum* in the peanut rhizosphere, thereby enhancing peanut drought tolerance [38]; *Tuber indicum* can mitigate drought-induced damage in the host plant by stabilizing the rhizosphere bacterial community structure of *Pinus armandii* and enhancing amino acid and sugar metabolism, thereby promoting the accumulation of the osmoprotectant proline [130]. Collectively, these studies indicate that under drought conditions, beneficial fungi can mediate the restructuring of the plant rhizosphere microbiome to better meet host needs. On this basis, we here summarize the major mechanisms by which beneficial fungi mediate rhizosphere microbiome restructuring. The assembly of plant microbiomes is often explained by the amplification–selection model: driven by rhizodeposits, certain microbial taxa first undergo pronounced numerical amplification in the rhizosphere, after which the host plant selects among these amplified microbes, ultimately shaping a host-specific rhizosphere microbiome [131]. Within this framework, the formation of a drought-protective rhizosphere microbiome can be summarized as being governed by three key drivers: (1) rhizodeposits; (2) plant immunity; and (3) microbe–microbe interactions.

#### 2.2.1. Rhizodeposits

Living roots release approximately 5–20% of photosynthetically fixed carbon into the soil [132,133]. Plants continuously deliver organic carbon to the belowground environment through root exudates, mucilage, and sloughed-off cells; this “frequent drip” of carbon inputs is collectively termed rhizodeposition [134,135]. Rhizodeposits are chemically diverse, comprising not only relatively simple inorganic components (e.g., bicarbonate and protons) but also a wide range of metabolites, including sugars, flavonoids, fatty acids, organic acids, amino acids, and proteins [136]. Under drought conditions, rhizodeposits can reshape rhizosphere microbiome composition, thereby influencing host growth and stress adaptation [137,138,139]. For example, under drought, *Ph. liquidambaris* B3 can modulate the flavonoid formononetin in rhizodeposits, and formononetin can enhance peanut drought tolerance by enriching drought-protective AMF communities [38]. Strigolactones in rhizodeposits facilitate the establishment of mutualistic symbiosis with AMF [140,141]. In rice, strigolactones are also associated with improved root development and enhanced drought tolerance [142,143]. In addition, benzoxazinoids can regulate microbial community structure in the maize endosphere and rhizosphere [144]. Collectively, these studies indicate that rhizodeposits promote direct plant–microbe communication and can act as signaling cues that drive the recruitment and restructuring of root-associated microbiota. Beyond signaling, integrated approaches combining microbiomics, comparative genomics, and metabolomics have revealed coupling between microbial substrate-utilization traits and rhizodeposit chemistry [145]. Fast-growing taxa (e.g., Proteobacteria) preferentially exploit readily degradable sugar substrates, whereas drought-adapted groups (e.g., Actinobacteria) tend to utilize structurally complex, more slowly degradable phenolic compounds [146]. Moreover, some bacteria can depolymerize complex organic molecules into simpler forms that can subsequently be consumed by other microbes [147]. Conversely, microbial metabolism of root exudates can alter the rhizosphere chemical milieu, which may in turn influence both the composition and quantity of plant exudates. Through plant metabolic feedbacks, these dynamics may either reinforce the initial microbial community or create ecological opportunities for the subsequent recruitment of additional microbial members [146].

#### 2.2.2. Plant Immunity

The plant immune system is a key determinant of rhizosphere microbiome assembly [148]. By restricting and selectively permitting microbial colonization, plants shape the structure and function of rhizosphere communities [149]. This process relies on the recognition of highly conserved microbe-associated molecular patterns (MAMPs) and the ensuing MAMP-triggered immunity (MTI), which constitutes a primary barrier limiting colonization [150]. Drought stress may attenuate immune responsiveness, thereby shifting microbiome composition and potentially facilitating the establishment of certain beneficial microbes [151]. In addition, crosstalk between drought-related hormonal signaling and immunity in roots can further influence root-associated microbial communities [103,152]. Under drought, host roots ABA, and ABA signaling can antagonize SA-mediated immune pathways, suggesting that a partial reduction in immune sensitivity may be advantageous for roots under water deficit [153,154]. Although the direct effects of root ABA on microbiome composition remain incompletely resolved, evidence indicates that ABA-activated plant genes can induce ROS production in the apoplast, where most microbes reside [155,156]. ROS dynamics have been linked to drought-associated shifts in the root microbiome and may thereby contribute to improved host drought tolerance [157]. Meanwhile, the plant microbiome can be viewed as an “additional component” of the plant immune system, as microbe–microbe interactions within the community can reduce the risk of disease outbreaks under drought [150]. Collectively, plant immunity and the microbiome interact bidirectionally and cooperate to support host performance under drought stress.

#### 2.2.3. Microbe–Microbe Interactions

Microbe–microbe interactions also contribute to the assembly of rhizosphere-associated microbiomes [150,158]. Wang et al. constructed a highly simplified sorghum rhizosphere syncom to test how microbial interactions influence community assembly under drought. Using a strain-by-strain removal approach, they found that deleting only *Rhizobium* sp. 4F10 was sufficient to trigger syncom collapse and further weaken its drought-protective function, indicating that this strain may act as a keystone member during community assembly [159]. In addition, by profiling bacteria, fungi, and oomycetes across different compartments in diverse *Arabidopsis thaliana* genotypes, researchers built interkingdom interaction networks and identified two key microorganisms as “mediators” linking abiotic factors, host determinants, and the colonization of other microbial members. Removing one or more strains from the syncom caused pronounced shifts in community structure; however, once the community was established, reintroducing the removed strains did not substantially alter community composition, suggesting that priority effects operate mainly during the initial colonization phase [160,161]. Microbial interactions not only affect community stability but can also enhance plant drought resistance. For example, rhizosphere fungal communities associated with the herbaceous grass *Panicum hallii* can restructure bacterial community composition, thereby helping plants withstand drought stress [162].

## 3. Conclusions and Future Prospects

**Figure 1:**
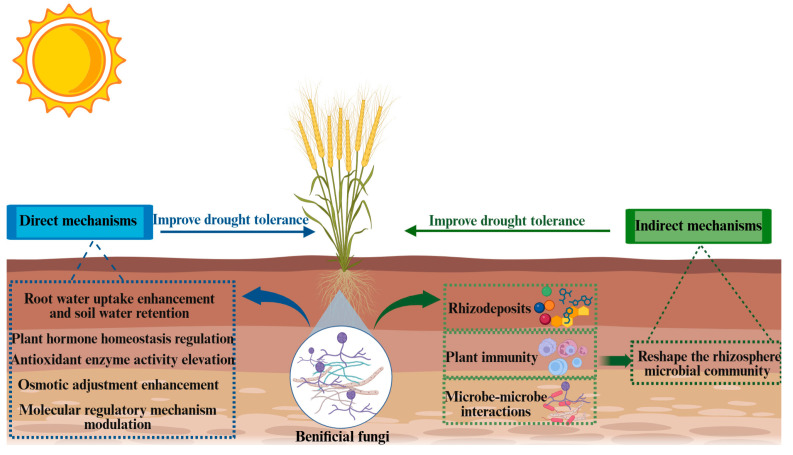
Plant-beneficial fungi primarily enhance crop drought tolerance via two mechanisms. (1) Direct mechanisms: beneficial fungi coordinately improve host drought resistance by modulating physiological and molecular responses, including enhancing root water uptake and soil water retention, regulating plant hormone homeostasis, elevating antioxidant enzyme activities, strengthening osmotic adjustment, and modulating drought-responsive molecular regulatory networks. (2) Indirect mechanisms: beneficial fungi can alter the quantity and composition of rhizodeposits and, together with plant immune regulation and microbe–microbe interactions, drive rhizosphere microbiome reassembly, thereby enriching drought-protective microbial communities. Blue boxes and arrows represent the direct mechanisms, whereas green boxes and arrows represent the indirect mechanisms.

In this review, we summarize the impacts of drought stress on crop growth and the key mechanisms by which plant-beneficial fungi mitigate the adverse effects of drought through crop physiology, molecular mechanisms, and rhizosphere microbiome reassembly. Plant-beneficial fungi enhance host drought tolerance mainly through two pathways: direct mechanisms (modulating physiological and molecular responses) and indirect mechanisms (reshaping the rhizosphere microbial community). Within this context, we highlight the key roles of rhizodeposits, plant immune regulation, and microbe–microbe interactions in the beneficial fungus-mediated restructuring of the rhizosphere microbiome (Figure 1).

Although substantial progress has been made in recent years in understanding how plant-beneficial fungi enhance crop drought tolerance, significant knowledge gaps remain in the systematic identification, quantitative characterization, and functional attribution of rhizodeposits across crop species and drought scenarios. Because roots can release hundreds of structurally diverse primary and secondary metabolites, while microbes simultaneously produce a broad array of compounds, distinguishing plant- versus microbe-derived signals and establishing their causal roles remain key bottlenecks. Accordingly, future research should prioritize: constructing standardized, cross-species rhizodeposit atlases under gradients of drought intensity and duration, coupled with time-resolved sampling to capture dynamic changes; integrating isotope tracing (e.g., plant carbon labeling), rhizosphere–soil metabolomics, and targeted validation to strengthen source attribution and causal inference; and leveraging simplified experimental platforms (e.g., gnotobiotic systems and microcosms), together with genome- and trait-informed modeling, to establish predictive links between key rhizodeposit cues and microbiome assembly and functional outcomes.

From a translational perspective, microbial inoculants are increasingly viewed as a promising route to enhance crop drought resilience by combining environmental compatibility with agricultural sustainability. However, converting rhizodeposit-informed knowledge into deployable inoculant products requires explicit community design rules. We therefore recommend developing rule-based syncom design pipelines that integrate strain–strain interaction analyses with assessments of plant stress-related traits (e.g., water-use efficiency, root hydraulic properties, ROS homeostasis, and antioxidant defense capacity). In parallel, multi-site and multi-season field trials should be prioritized to quantify effect sizes and failure thresholds, while simultaneously tracking syncom establishment and persistence and its impacts on native microbiomes and soil functions. Collectively, these efforts will facilitate the identification of more stable and deployable beneficial microbial communities for drought regions, thereby supporting yield stability and soil health improvement.

## Figures and Tables

**Table 1:** Effects of beneficial fungi on crop drought resistance and direct mechanisms.

Crop	Microbes	Related Mechanisms					References
		Root Water Uptake and Soil Water Retention	Hormone Homeostasis	Antioxidant Enzyme	Osmotic Adjustment	Molecular Mechanisms	
Peanut	*Gigaspora margarita*	√		√	√		[67,68]
	*Ph. liquidambaris* B3 and *Claroideoglomus etunicatum*		√	√	√		[38]
Tomato	*Rhizophagus irregularis*		√			√	[69,70]
	*Aspergillus violaceofuscus* and *Bacillus licheniformis*				√		[71]
Orange	*Funneliformis mosseae*			√	√	√	[72,73,74]
*Myrtus communis* L.	*Funneliformis mosseae*, *Rhizophagus irregularis* and *Pseudomonas fluorescens*			√			[75]
Soybean	*Funneliformis mosseae* and *Pseudomonas putida*		√				[76]
	*Glomus mosseae*	√			√		[77]
	*Piriformospora indica*		√			√	[78]
Maize	*Aspergillus oryzae* and *Aspergillus fumigatus*		√	√	√	√	[79]
	*Acaulospora scrobiculata*, *Paraglomus occultum*, *Rhizophagus intraradices*, *Glomus versiforme*, *Funneliformis mosseae*, *Claroideoglomus etunicatum*		√	√	√	√	[80,81]
	*Bacillus megaterium* and *Rhizophagus irregularis*	√			√	√	[82]
*Robinia pseudoacacia*	*Rhizophagus irregularis*			√		√	[83,84,85]
Rice	*Trichoderma harzianum*					√	[86]
	*Azospirillum brasilense* and *Glomus intraradices*			√	√		[87]
	*Funneliformis mosseae*, *Funneliformis geosporum*, *Claroideoglomus claroideum*, *Glomus microaggregatum*, *Rhizophagus irregularis*		√				[88]
Wheat	*Trichoderma harzianum*					√	[89]
	*Piriformospora indica*		√			√	[90]
	*Rhizophagus irregularis* and *Funneliformis mosseae*					√	[91]
*Medicago sativa*	*Rhizophagus irregularis*					√	[92]

Note: “√” indicates that the corresponding references involve, respectively, root water uptake and soil water retention, hormone homeostasis, antioxidant enzymes, osmotic adjustment, and molecular mechanisms.

## Data Availability

No new data were created or analyzed in this study. Data sharing is not applicable to this article.
